# An angiographic study on the morphometry of the anterior communicating artery complex- a South African sample

**DOI:** 10.1007/s00276-025-03670-4

**Published:** 2025-06-13

**Authors:** Mbalentle Madolo, Geney Gunston, Stuart More, Kentse Mpolokeng

**Affiliations:** 1 Division of Clinical Anatomy and Biological Anthropology, Department of Human Biology, University of Cape Town, Cape Town, South Africa; 2 Division of Nuclear Medicine, Department of Radiation Medicine, Groote Schuur Hospital, Cape Town, South Africa

**Keywords:** Anterior communicating artery complex, Anterior cerebral artery, Anterior communicating artery, Morphometric parameters

## Abstract

**Purpose:**

The anterior communicating artery complex (ACAC) comprises the A1 and A2 segments of the anterior cerebral artery (ACA) and the anterior communicating artery (ACoA). It is a common site for aneurysms and variations in anterior cerebral circulation. With ongoing advancements in endovascular intervention, there is an increasing need for detailed anatomical knowledge of the ACAC. This study was conducted to explore the anatomical measurements, including the length and diameter, of the ACAC in a South African population.

**Method:**

The study involved the analysis of 200 adult magnetic resonance angiogram (MRA) scans (145 female, 55 male) from Groote Schuur Hospital, Department of Radiology, where the internal length and diameter of ACAC vessels were obtained.

**Results:**

The average internal length of the right and left A1 ACA segments was 13.5 (3.48) mm and 12.85 (2.57) mm, respectively. The internal diameter of the right and left ACA was 2.26 (0.45) mm and 2.25 (0.68) mm, respectively. For the A2 ACA, the average internal length for the right A2 ACA was 23.4 (3.94) mm, and the left A2 ACA had an average internal length of 23.7 (3.97) mm. The average internal diameter for the right and left A2 ACA was 2.3 (0.33) mm and 2.37 (0.78) mm, respectively. For ACoA, the average internal length was 1.5 (1.76) mm, and the average diameter was 0.85 (0.91) mm.

**Conclusion:**

The morphometric parameters of the ACAC measured in this study were within similar ranges to those reported in previous international and national studies.

## Introduction

Blood supply to the human brain may be divided into the anterior and posterior circulations [22]. Although connected, these circulations are supplied by different arteries that perfuse the brain with blood in other regions of the cerebral mass [11, 13]. The left and right internal carotid arteries (ICA) and the right and left vertebral arteries are considered to be the two sources of blood supply to the brain [22]. It is these arteries that give rise to cerebral arteries that form a crucial arterial ring at the base of the brain, referred to as the Circulus arteriosus cerebri (CAC) [13,]. The CAC encircles the middle area of the brain. It is located in the region of the hypothalamus and cerebral peduncles encircling the pituitary gland [11, 13]. This anastomosis is from which the anterior and posterior circulations are derived. The brain’s anterior circulation supplies blood to most cerebral hemispheres, including the frontal and parietal lobes, lateral temporal lobes, and anterior parts of deep cerebral hemispheres [18, 30]. This circulation consists of the bilateral ICA, which both divide into terminal branches: the anterior cerebral artery (ACA), the middle cerebral artery (MCA) and the anterior communicating artery (ACoA) [6, 17]. Due to the ring-like nature of the CAC, this anterior circulation will join the posterior circulation through the posterior communicating arteries (PCoA) [26]. Within the anterior circulation, a crucial complex referred to as the anterior communicating artery complex (ACAC) is reported to have significant clinical relevance as it is the most common site of intracranial aneurysms. More specifically, the ACoA is said to be the most frequent site of cerebral aneurysms [22]. Additionally, many studies have highlighted that ACA A1 segment variations are the most frequent anomalies that accompany ACoA aneurysms [12].

This complex consists of the proximal ACA A1 segment of the ACA, the A2 segment of the ACA, and the ACoA [23]. Additionally, several studies have reported a high frequency of variation in the anterior cerebral circulation, specifically in the A1 segment, A2 segment, and ACoA It has been shown that asymmetry of the A1 segments of the ACAC has a significant correlation with cerebral aneurysm formation at the junction of the dominant A1 segment and the ACoA or directly in the ACoA [3]. It is hypothesised that it is the increased blood flow in the vessel with a larger diameter, which in turn increases the hemodynamic stress, that is responsible for this aneurysm formation [3, 22]. Using glass models of the ACAC, a direct relationship between increased asymmetry of the A1 segments of both ACAs or increased flow in one of the two A1 segments and increased hemodynamic wall shear stress on the ACoA, causing intracranial aneurysms was shown [30].

While this complex has considerable clinical importance, there is limited knowledge about the morphological variability and morphometric parameters of the arteries of the ACAC [23]. A high aneurysm rupture risk has been associated with anterior circulation aneurysms [9]. ACAC aneurysms have been reported to be the most likely to rupture when compared to aneurysms that may be present elsewhere in the CAC [16]. Therefore, gaining comprehensive knowledge about ACAC anatomy will be of great use to clinicians and neurosurgeons to diagnose and prevent cerebral conditions such as aneurysms and strokes.

Furthermore, amongst other modalities for treating intracranial aneurysms, endovascular coiling has become the primary intervention used by vascular neurosurgeons [23]. Although this procedure has become popular because of its minimally invasive nature and reduction in risk during procedures in comparison to other modalities, unfavourable aneurysm formation and unexpected anatomical variations of vessels still pose significant challenges [23]. The constant advances in various aspects of endovascular intervention have highlighted the importance of sufficient literature pertaining to the anatomical knowledge of the ACAC [7, 18].

As of 2018, cerebrovascular diseases are ranked third amongst the top ten leading natural causes of mortality in South Africa [28]. Thus, this further underscores the necessity for a population-based study detailing the anatomical parameters of the vessels of the ACAC within a South African population. Given the observed connection between the anatomy of the proximal ACA (comprising the A1 and A2 segments) and ACoA aneurysms, this study primarily aimed to describe the morphometric parameters of the proximal ACA (A1 and A2 segment) and ACoA, displaying typical anatomy, within a South African sample. Additionally, this study investigated the relationship between these morphometric parameters and demographic factors (sex and age).

## Materials and methods

Morphometric analysis of retrospective magnetic resonance angiographic (MRA) data was the method employed in this study. A descriptive, cross-sectional, observational study was completed on 200 three-dimensional (3D) time-of-flight (TOF) MRA images. The sample population included MRA images from individuals who underwent CAC MRA imaging at Groote Schuur Hospital between 1 January 2018 and 1 January 2023. The internal diameter and internal length of the A1 segment, A2 segment and ACoA were measured, and all results were recorded. To examine the anterior cerebral circulation in brain MRA images, unenhanced time-of-flight (TOF) 3D images were used. The volume rendering function of the picture archiving and communication digital imaging system (PACS) of the Department of Radiology, Groote Schuur Hospital, was used on the 3D TOF MRA images for optimum 3D visualisation of the arteries of the ACAC. Each image was analysed in multiplanar reformation (MPR) mode to assess various dimensions and planes of the brain. The axial and sagittal planes of MRA images showing typical anatomy were selected to measure the diameters and lengths of the A1 segment, A2 segment, and ACoA. The CAC was identified by navigating through different slices of the brain, with the axial view chosen for optimal visualization of the A1 segment and ACoA and the sagittal view selected for the visualisation of the A2 segment. Once the CAC was located, the ACAC was identified by zooming into the anterior part of the CAC. Each artery was measured twice for its internal length, and the average of the two measurements was calculated. For the internal diameters, a single measurement was taken at three different points along the artery, and the average value was then determined. For this study, only images with a three-dimensional time of flight (3D-TOF) sequence were used. MRA images that had severe susceptibility artefact presence that obscured or hindered the visualisation of the arteries were excluded. ACAC arteries that exhibited non-typical anatomy were excluded from the study. Furthermore, ACAC arteries that were obscured or compromised by the presence of endovascular coiling or other surgical intervention were also excluded from the study. After exclusion criteria was applied for each artery, only 183 MRA images were used for A1 ACA measurements, 181 MRA images for A2 ACA measurements and 46 MRA images were utilised for ACoA measurements.

**Fig. 1:**
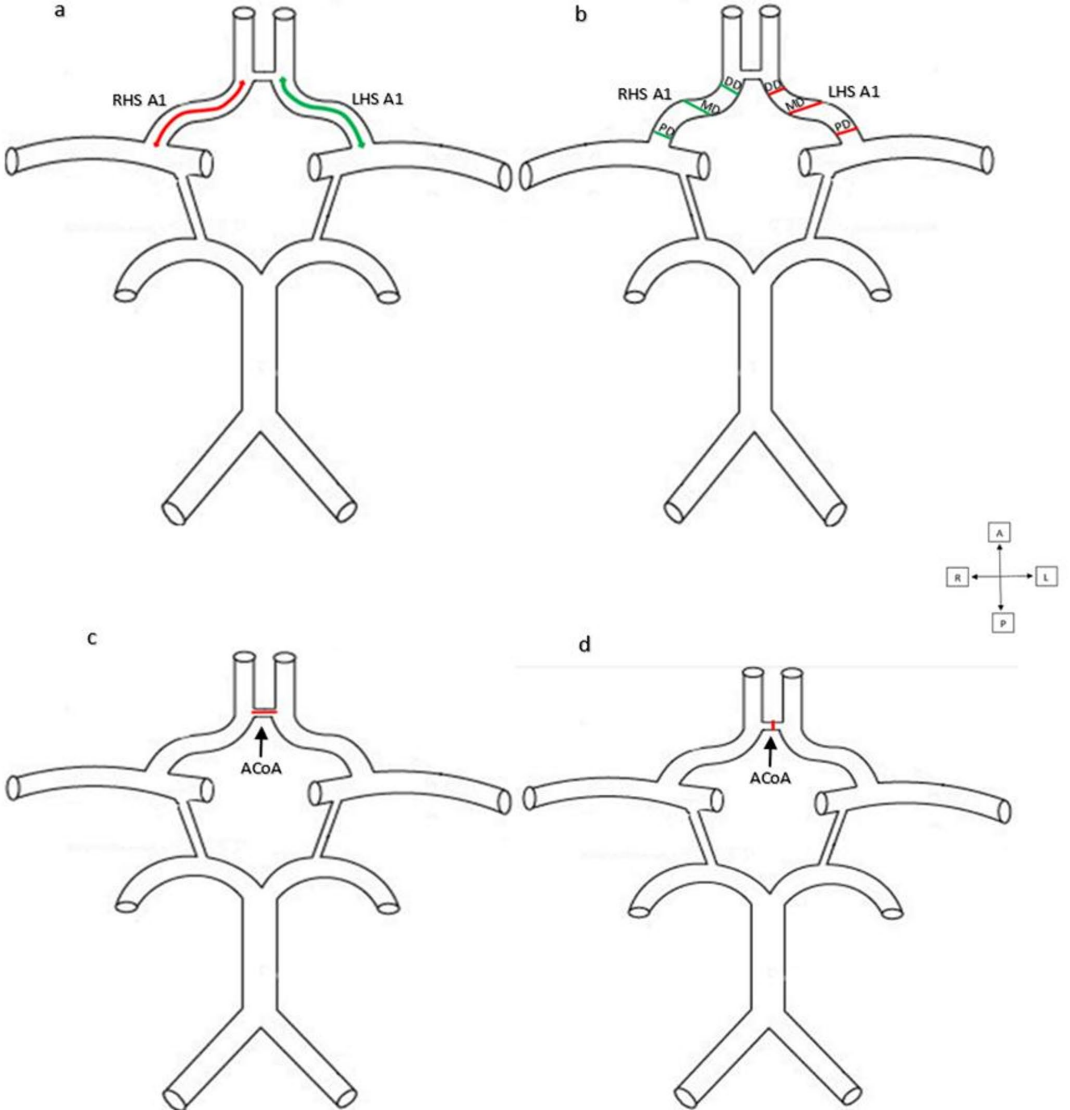
Diagram indicating where the measurements of the internal length and internal diameter of the right A1 segment, left A1 segment, and the anterior communicating artery were taken on magnetic resonance angiograms

**Fig. 2:**
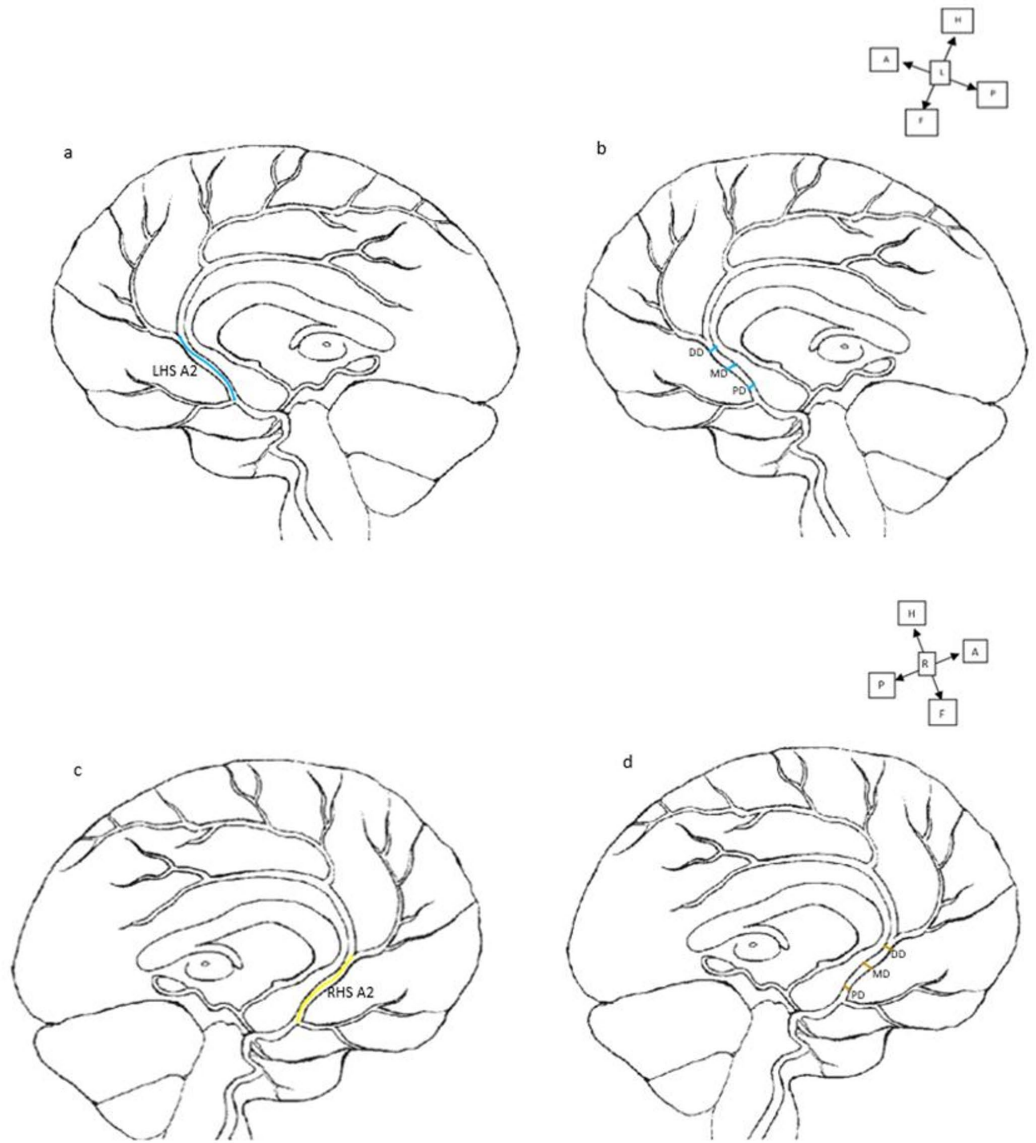
Diagram indicating where the internal length and internal diameter measurements of the right and left A2 segments were taken on magnetic resonance angiograms. **a** internal length of the left A2 segment (blue line), **b** internal diameters of the left A2 segment, **c** internal length of the right A2 segment (yellow line) and **d** internal diameters of the right A2 segment, A2- A2 segment of the anterior cerebral artery, DD- distal diameter region, MD- middle diameter region, PD- proximal diameter region, A-anterior, L-left, R-right, P-posterior, H- head, F-foot

As illustrated in Figs. 1 and 2 below, the straight distance and cross-path measurement tools on PACS were used to measure the internal diameters and lengths of the A1 segment, A2 segment, and ACoA, based on anatomical landmarks. Length measurements were taken as follows:

- ACA (A1 segment): Measurements were taken from the ICA terminal bifurcation to the origin of the ACoA.
- ACA (A2 segment): Measurements were taken from where the ACoA connects to the ACA on both sides to the junction of the rostrum and genu of the corpus callosum, or the origin of the callosomarginal artery.
- ACoA: Measurements were taken between the left and right ACAs.

Diameter measurements were conducted as follows:

The internal diameter of each artery was measured at three points perpendicular to the long axis of the artery and averaged for accuracy. These points were labelled as the proximal diameter region (PD), middle diameter region (MD), and distal diameter region (DD).

- PD: Measurements were taken closest to the artery’s origin.
- MD: Measurements were taken approximately halfway between the artery’s origin and termination (i.e., between PD and DD).
- DD: Measurements were taken closest to the artery’s termination.

(a) internal length of the right (red line) and left (green line) A1 segments, (b) internal diameters of the right and left A1 segments, (c) internal ACoA length and (d) internal ACoA diameter, RHS- right-hand side, LHS- left-hand side, A1- A1 segment of the anterior cerebral artery, ACoA- anterior communicating artery, DD- distal diameter region, MD- middle diameter region, PD- proximal diameter region, A-anterior, L-left, R-right, P-posterior.

The measurements were recorded on a formulated Microsoft Excel datasheet. Digital photographs of the MRA images were also taken using the Microsoft Word screenshotting function. Statistical analysis was performed on the recorded data transferred to REDCap software and was loaded onto the statistical package for the social sciences IBM© programme (SPSS). SPSS was used to determine the statistical significance of the data collected in this study. The data distribution was tested with the data set considered to be normally distributed if the Shapiro-Wilk test reported a p-value > 0.05 and with kurtosis and skewness between − 1 and + 1. All the qualitative data was tested for normality using the Shapiro-Wilk test. The measurements are reported as the median value and interquartile range in parenthesis for the data that was not normally distributed. The data underwent further descriptive analysis, allowing for more information, such as median, mean, interquartile range, standard deviations and variance. To analyse if the measurements differed between sex and age groups, correlation tests such as the Mann-Whitney U test or Kruskal-Wallis test analysis were completed.

## Results

### Demographic characteristics

From the 200 MRA images observed, 72.5% were female, and 27.5% were male. The sample was further categorized according to age cohorts as follows: <30 (3.37%); 31–40 (12.98%); 41–50 (28.37%); 51–60 (26.44%); 61–70 (20.19%); 71–80 (5.29%) and > 81 (3.37%). The mean age in the angiographic cohort was 52.84 (± 13.17) years old, with the minimum age recorded at 17 years old and the maximum age at 84 years old. The Shapiro-Wilk test was used to test for normality. The age of the patients was normally distributed with a p-value of 0.359.

### A1 segment of ACA and A2 segment of ACA parameters

The average internal length of the right and left A1 ACA segments was 13.5 (3.48) mm and 12.85 (2.57) mm, respectively. Additionally, the internal diameter of the right and left ACA was 2.26 (0.45) mm and 2.25 (0.68) mm, respectively. For the A2 ACA, the average internal length for the right A2 ACA was 23.4 (3.94) mm, and the left A2 ACA had an average internal length of 23.7 (3.97) mm. The average internal diameter for the right and left A2 ACA was 2.3 (0.33) mm and 2.37 (0.78) mm, respectively.

Using the Wilcoxon Signed Rank test, the parameters of the right A1 segment and A2 segment and the adjacent left A1 and A2 segment were compared to assess whether there was a significant difference between the measurement on each side (Right vs. Left). As seen in Table 1, all the p-values were greater than 0.05, thuswe can reject the hypothesis that there is a significant difference in morphometric parameters between the right A1 and A2 segment and the left A1 and A2 segment in the MRA images used for this study.

**Table 1:** Internal length and internal diameter measurements of the A1 and A2 segments from MRA images

ACAC Measurements (mm)	Left	Right	*P*-Value
A1 Length	12.85 (2.57)	13.5 (3.48)	0.41
A1 Diameter	2.25 (0.68)	2.26 (0.44)	0.361
A2 Length	23.7 (3.97)	23.4 (3.94)	0.059
A2 Diameter	2.37 (0.78)	2.3 (0.33)	0.347

### Parameters of ACoA

**Table 2:** Median length and diameter of the anterior communicating artery from the MRA images

ACoA Measurements (mm)	MED (IQR)
ACoA Length	1.5 (1.76)
ACoA Diameter	0.85 (0.91)

As seen in Table 2, the average internal length of ACoA was 1.5 (1.76) mm and the average diameter was 0.85 (0.91) mm.

### Morphometric parameters of the ACAC with respect to sex and age

**Table 3:** Morphometric parameters of the anterior communicating artery complex

		RHS A1 Length (mm)	LHS A1 Length (mm)	RHS A1 Diameter (mm)	LHS A1 Diameter (mm)	RHS A2 Length (mm)	LHS A2 Length (mm)	RHS A2 Diameter (mm)	LHS A2 Diameter (mm)	ACOA Length (mm)	ACOA Diameter (mm)
Median (IQR)		13.5 (3.48)	12.85 (2.57)	2.26 (0.45)	2.25 (0.68)	23.4 (3.94)	23.7 (3.97)	2.3 (0.33)	2.37 (0.78)	1.5 (1.76)	0.85 (0.91)
Sex	Male	13.95 (3.3)	13.3 (2.65)	2.4 (0.2)	2.4 (0.2)	24 (3.53)	24.4 (3.69)	2.4 (0.29)	2.5 (0.28)	0 (2)	0 (0.96)
	Female	13.3 (3.5)	12.85 (2.5)	2.26 (0.44)	2.23 (0.76)	23.4 (4.07)	23.7 (4.05)	2.3 (0.35)	2.4 (0.89)	1.8 (1.7)	1.05 (0.88)
	*P*-value^a^	0.105	0.223	0.022	0.342	0.259	0.095	0.11	0.219	0.284	0.134
Age	<30	13.9 (6.69)	12.2 (4.04)	2.17 (0.16)	2.35 (2.9)	25.9 (3.03)	26.25 (5)	2.27 (0.97)	2.63 (0.67)	3.7 (1.66)	1.5 (1.17)
	31-40	13.25 (3.8)	13.25 (2.81)	2.4 (0.37)	2.3 (0.42)	26.45 (4.2)	24.55 (3.99)	2.3 (0.23)	2.57 (1.54)	1.75 (1.62)	0.4 (0.91)
	41-50	13.2 (4.12)	13.05 (2.64)	2.35 (0.33)	2.35 (0.33)	23.1 (3.5)	23.2 (3.92)	2.37 (0.27)	2.37 (0.28)	2.13 (1.8)	0.85 (0.84)
	51-60	13.35 (3.2)	13.1 (2.42)	2.28 (0.48)	2.2 (0.32)	22.95 (4.38)	24.3 (3.8)	2.3 (0.33)	2.2 (0.99)	1.1 (1.77)	0.9 (0.96)
	61-70	12.9 (2.3)	12.1 (2.4)	2.38 (0.63)	2.35 (0.58)	24.15 (3.7)	25.1 (4.22)	2.38 (0.33)	2.27 (0.32)	1.3 (1.7)	1.12 (0.84)
	71-80	14.1 (2.63)	13.8 (2.18)	2.17 (0.21)	2.15 (0.13)	21.58 (3.9)	23.5 (4.4)	2.37 (0.11)	2.25 (0.17)	0 (1.2)	0 (1.07)
	81>	13.9 (2.5)	13.1 (2.7)	2.07 (0.13)	2.1 (0.32)	25.2 (4.1)	25.9 (4.2)	2.4 (0.11)	2.4 (0.3)	0 (1.2)	0 (0.96)
	*P*-value^b^	0.757	0.783	0.229	0.299	0.233	0.825	0.779	0.006	0.107	0.764

a− These *P*−values were obtained by running a Mann−Whitney U test

b− These *P*−values were obtained by running a Kruskal−Wallis test

Table 3 shows that all the *P*-values were greater than 0.05 except for the RHS A1 diameter, which reported a p-value of 0.022. Therefore, the null hypothesis that there is no significant difference in A1 ACA length, A2 ACA length and ACoA length between females and males is accepted. However, the null hypothesis of no significant difference in RHS A1 segment diameter between females and males is rejected (*p* = 0.022). In Table 3, we can further see that females have a smaller RHS A1 diameter (2.26 (0.44) mm) than males (2.4 (0.2) mm). Additionally, Table 3 shows that when looking at the difference in the morphometric parameters in the ACAC for age groups, no significance was found except for the LHS A2 diameter (*p* = 0.006). The age group with the largest LHS A2 diameter was the ‘<30 years old’ age group. The ‘51–60 years old’ age group had the smallest diameter. The age group ’61–70 years old deviated from the pattern; however, between the ages of < 30 years old and 60 years old, a decrease in LHS A2 diameter can be seen. This study found no significance between A1 ACA length, A2 ACA length, and ACoA length.

## Discussion

Rupture of cerebral aneurysms not only causes subarachnoid haemorrhages (SAH) but leads to high mortality and morbidity [10]. The incidence of SAH lies between 10 and 36 per 100 000 people per year depending on geographical location [3]. Worldwide, approximately 500 000 individuals will experience SAH each year, with the majority of individuals being in low and middle-income countries [15]. According to a 2014 study conducted in South Africa, it was observed that among all the recorded intracranial aneurysms, the most prevalent cases were ACAC aneurysms, more specifically anterior communicating aneurysms [2, 18]. The substantial impact of cerebrovascular diseases on morbidity in South Africa emphasises the need for additional research and understanding in this field. The length and diameter of the ACAC are crucial as they can be used to investigate pathological conditions such as aneurysms and aid in the diagnosis and treatment interventions of various cerebrovascular diseases. Thus, this study aimed to investigate the anatomical dimensions, length and diameter of the ACAC in a South African sample.

**Table 4:** A summary of studies showing morphometric parameters of the ACA A1 and ACA A2

Author	Region	Sample (*n*)	Right A1 Length (mm)	Left A1 Length (mm)	Right A1 Diameter (mm)	Left A1 Diameter (mm)	*Right A2 Length (mm)	*Left A2 Length (mm)	*Right A2 Diameter (mm)	*Left A2 Diameter (mm)
Krishnamurthy et al. [21]	Spain	93	14.49 ± 0.28	14.22 ± 0.22	2.12 ± 0.07	2.32 ± 0.06	–	–	–	–
Aggarwal et al. [1]	India	120	15.78 ± 3.71	17.37 ± 4.84	–	–	–	–	–	–
Kedia et al. [19]	India	15	12.09 (10–15)	12 (10–15)	2.32 (2–3)	2.36 (1.5–3)	–	–	–	–
Karatas et al. [17]	Turkey	100	14.44 ± 2.32	13.72 ± 2.12	1.87 ± 0.48	1.96 ± 0.49	–	–	–	–
Cilliers et al. [5]	South Africa	61	14.11 ± 2.40	13.45 ± 2.09	–	–	–	–	–	–
Canaz et al. [4]	Turkey	30	13.56 ± 2.25	13.76 ± 1.87	1.54 ± 0.37	1.84 ± 0.36	18.83 ± 3.18	18.73 ± 3.02	1.86 ± 0.13	1.84 ± 0.015
Shatri et al. [27]	Greece	513	14.1 ± 1.51	13.87 ± 1.3	2.04 ± 0.28	2.06 ± 0.26	–	–	–	–
Dhanalakshmi et al. [8]	India	50	14.3 (11–18)	13 7 (9–18)	1.76 (0.068–2.23)	1.8 (0.53–2.57)	–	–	–	–
Hassan et al. [14]	Egypt	50	16.11 ± 3.4	15.83 ± 2.31	2.24 ± 0.33	2.18 ± 0.37	37.72 ± 3.33	37.93 ± 3.44	2.34 ± 0.62	2.27 ± 0.63
Luckrajh et al. [23]	South Africa	100	13.36 ± 2.70	12.43 ± 2.90	1.66 ± 0.47	1.77 ± 0.53	24.72 ± 6.12	25.07 ± 5.88	1.59 ± 0.42	1.6 ± 0.41
Present study	South Africa	183	13.5 (3.48)	12.85 (2.57)	2.26 (0.45)	2.25 (0.68)	23.4 (3.94)	23.7 (3.97)	2.3 (0.33)	2.37 (0.78)

*Total A2 sample (n) is 181

– Indicates the absence of measurement

In Table 4, the internal lengths and diameters of the ACAC arteries were recorded using either mean ± standard deviation (SD), mean (min-max), or median (IQR) in millimetres (mm), depending on the data distribution. This study presents all morphometric parameters as median (IQR), which is more appropriate for non-normally distributed data.

Literature indicates that the average length of the right A1 segment ranges from 12.09 mm to 16.11 mm, with various standard deviations and ranges reported. In this study, the right A1 segment length of 13.50 (3.80) mm falls within this range. Canaz [4] reported a length of 13.56 ± 2.25 mm for the right A1 segment, which is very similar to our finding. Cilliers [5], from South Africa, reported a slightly higher value of 14.11 ± 2.40 mm. Another South African study by Luckrajh [23], which had a smaller sample size but focused on patients with ACoA aneurysms, reported a length of 13.36 ± 2.70 mm, close to our results.

The left A1 segment length has a wider range of 12.07–17.37 mm. In this study, the average length of the left A1 segment is 12.85 (2.57) mm, which is within the reported range. Cilliers [5] found a length of 13.45 ± 2.09 mm, while Luckrajh [23] reported 12.43 ± 2.90 mm, more closely aligned with our results. The diameter of the right A1 segment in this study is 2.26 (0.45) mm, fitting within the range of 1.66 mm to 2.32 mm. The diameter for the left A1 segment ranges from 1.77 mm to 2.36 mm, with our result of 2.25 (0.68) mm also within this range. Hassan [14] reported similar diameters for both segments: 2.24 ± 0.33 mm for the right A1 and 2.18 ± 0.37 mm for the left A1. Table 4 shows a broad range of measurements for both right and left A2 segment lengths. The right A2 segment ranges from approximately 12.09 mm to 37.93 mm, and the average length in this study is 23.4 (3.94) mm, which fits within the previously reported range. For the left A2 segment, the range is 11.63 mm to 37.93 mm, with our study reporting 23.7 (3.7) mm, also consistent with the literature. The large range in A2 segment lengths may be due to differences in defining the origin and termination of the A2 segment and methodological variations across studies. The left A2 diameter in this study, 2.37 (0.78) mm, does not fall within the range of ± 1.6 mm to 2.27 mm shown in Table 4.

Table 5 Shows that the ACoA length recorded in the present study is within the range provided by the previous studies. However, when strictly comparing the mean and median, the ACoA diameter for this study does not fall within the range provided by previous studies

**Table 5:** A summary of morphometric parameters of the anterior communicating artery

Author	Region	Sample (*n*)	ACOA Length	ACOA Diameter
Murray et al. [24]	Australia	35	0.5	1
Cilliers et al. [5]	South Africa	61	3 ± 1.33	–
Shatri et al. [27]	Greece	513	2.99 ± 0.62	1.16 ± 0.17
Tripathi et al. [29]	India	100	2.80 (1.5–5.9)	1.11 (0.59–2.1)
Present study	South Africa	46	1.5 (1.76)	0.85 (0.91)

– Indicates the absence of measurement

To test whether there is a difference in the measurements of the ACAC parameters about sex (female vs. male), the Mann-Whitney U test was used. In the present study, the null hypothesis that there is no significant difference in A1 ACA length, A2 ACA length and ACoA length between females and males is accepted. Contrarily, Sharma [26] observed that ACA length is longer in females than in males. Sharma [26] concluded that the males’ general ACA diameter is smaller. However, previous studies from Krabbe-Hartkamp [20], Aggarwal [1] and Shatri [27] all concluded that the general ACA diameter is larger in males than in females. The latter finding was reflected in the present study only concerning RHS A1 ACA diameter. A difference in RHS A1 ACA diameter between females and males was shown (*p* = 0.022). Additionally, this study showed that females have a smaller RHS A1 ACA diameter (2.26 (0.44) mm) compared to males (2.4 (0.2) mm). However, Sharma [26] concludes that the general ACA diameter is smaller in males.

In this study, the Kruskal-Willis test showed that when looking at the difference of the morphometric parameters in the ACAC with respect to age groups, no significance was found except for the LHS A2 diameter (*p* = 0.006) (Table 3). The age group with the largest A2 diameter was those under 30 years old, while the smallest diameter was observed in the 51–60 years old group. Therefore, there is a noticeable decrease in LHS A2 diameter from age 30 to age 80. Similarly, evidence from a study by Sharma [26] showed that ACA diameter is greater in younger age groups (< 40 years old). No significance was found between A1 ACA length, A2 ACA length and ACoA length in the present study. Conversely, Sharma [26] concluded that ACA length is larger in younger age groups (< 40 years old). There were a few limitations present in this study. Although the South African population reportedly has more females than males, the number of MRA scans used in this study from female patients outnumbered those from male patients by a very large margin. Thus, measurements related to sex were subject to sampling bias. For future studies, a sample that closely reflects the true representation of the South African public would be advisable. The present study used one type of imaging modality, three-dimensional TOF MRA (3D TOF MRA), which can be adversely affected by signal loss due to spin dephasing when blood flow is too slow or not properly aligned with the slice plane. Several MRA scans were compromised in this study, failing to meet the inclusion criteria due to susceptibility artifacts. Therefore, it is recommended that future research consider using additional imaging modalities alongside 3D TOF MRA.

## Conclusion

This study aimed to expand the limited research on the morphometric parameters of ACAC arteries within a South African sample. The measurements obtained are consistent with those reported in international studies and closely align with findings from previous research conducted in South Africa. These factors are crucial for investigation as they could be valuable in clinical settings, such as during revascularisation surgeries. Additionally, no significant difference in ACAC morphometry was observed between the cerebral hemispheres.

## Data Availability

The data that support the findings of this study are not openly available due to reasons of sensitivity and are available from the corresponding author upon reasonable request. Data are located in controlled access REDCap data storage.

## Abbreviations

3D: TOF Three-dimensional time-of-flight
An: Artery
ACA: Anterior cerebral artery
ACAC: Anterior communicating artery complex
ACoA: Anterior communicating artery
CAC: Circulus arteriosus cerebri
DD: Distal diameter
HREC: Human Research Ethics Committee
ICA: Internal carotid artery
MCA: Middle cerebral artery
MD: Middle diameter
MPR: Multiplanar reformation
MRA: Magnetic resonance angiogram
MRI: Magnetic resonance image
PACS: Picture archiving and communication digital imaging system
PCA: Posterior cerebral artery
PCoA: Posterior communicating artery
PD: Proximal diameter
POPIA: Protection of personal information act
REDcap: Research electronic data capture
SAH: Subarachnoid haemorrhages
SPSS: Statistical package for the social sciences
TOF: Time-of-flight

## References

[1] Aggarwal DN, Paul M, Mukherjee M (2022) Diameter of anterior cerebral artery on MRI angiograms. Int J Anat Res 4:2245–2250. 10.16965/ijar.2016.189

[2] Blignaut G, Loggenberg E, De Vries C (2014) The radiological appearance of intracranial aneurysms in adults infected with the human immunodeficiency virus (HIV). South Afr J Radiol 18(1). 10.4102/sajr.v18i1.586

[3] Burlakoti A, Kumaratilake J, Taylor DJ, Henneberg M (2020) Quantifying asymmetry of anterior cerebral arteries as a predictor of anterior communicating artery complex aneurysm. BMJ Surg Interv Health Technol 2:e000059. 10.1136/bmjsit-2020-000059 35047797 10.1136/bmjsit-2020-000059 PMC8749284

[4] Canaz H, Arslan M, Hacıoglu H, Tokmak M, Canaz G, Cavdar S (2018) Morphometric analysis of the arteries of Willis polygon. Romanian Neurosurg 32:56–64. 10.2478/romneu-2018-0007

[5] Cilliers K, Page BJ (2017) Description of the anterior cerebral artery and its cortical branches: variation in presence, origin, and size. Clin Neurol Neurosurg 152:78–83. 10.1016/j.clineuro.2016.11.024 27936431 10.1016/j.clineuro.2016.11.024

[6] Cilliers K, Page BJ (2015) Review of the anatomy of the distal anterior cerebral artery and its anomalies. Turk Neurosurg 26(5):653–661. 10.5137/1019-5149.JTN.14294-15.1 10.5137/1019-5149.JTN.14294-15.1 27337235

[7] Clarke H, Nefale T, Mngomezulu V (2023) Endovascular management of intracranial aneurysms at Chris Hani Baragwanath academic hospital. SA J Radiol 27:2634. 10.4102/sajr.v27i1.2634 37292418 10.4102/sajr.v27i1.2634 PMC10244967

[8] Dhanalakshmi V, Suresh Kumar T, Arun Kumar K, Sathish Kumar S (2019) Anterior cerebral artery: an anatomical study. Int J Anat Res 7:6494–6498. 10.16965/ijar.2019.149

[9] Dhar S, Tremmel M, Mocco J, Kim M, Yamamoto J, Siddiqui AH, Hopkins LN, Meng H (2008) Morphology parameters for intracranial aneurysm rupture risk assessment. Neurosurgery 63:185–197. 10.1227/01.NEU.0000316847.64140.81 18797347 10.1227/01.NEU.0000316847.64140.81 PMC2570753

[10] D’Souza S (2015) Aneurysmal subarachnoid hemorrhage. J Neurosurg Anesthesiol 27:222–240. 10.1097/ANA.0000000000000130 25272066 10.1097/ANA.0000000000000130 PMC4463029

[11] Du Toit F (2015) Circulus Arteriosus Cerebri: Anatomical variations and their correlation to Cerebral Aneurysms [Thesis]. University of Cape Town, Faculty of Health Sciences, Division of Clinical Anatomy and Biological Anthropology. http://hdl.handle.net/11427/16481

[12] Gunna SA, Wabale RN (2013) Variations of anterior cerebral artery in human cadavers. Neurol Asia 18(3):249–259

[13] Gupta G (2022) Circle of Willis Anatomy: Overview, Gross Anatomy, Natural Variants. Available via MedScape. https://emedicine.medscape.com/article/1877617

[14] Hassan N, Mansor M, Ibrahim A, Ibrahim I (2020) Anatomical measurements of cerebral arteries using digital Subtraction angiography. Ain Shams Med J 71:259–267. 10.21608/asmj.2020.125617

[15] Hughes JD, Bond KM, Mekary RA, Dewan MC, Rattani A, Baticulon R, Kato Y, Azevedo-Filho H, Morcos JJ, Park KB (2018) Estimating the global incidence of aneurysmal subarachnoid hemorrhage: A systematic review for central nervous system vascular lesions and Meta-Analysis of ruptured aneurysms. World Neurosurg 115:430–447. 10.1016/j.wneu.2018.03.220 29649643 10.1016/j.wneu.2018.03.220

[16] Jirjees S, Htun ZM, Aldawudi I, Katwal PC, Khan S (2020) Role of morphological and hemodynamic factors in predicting intracranial aneurysm rupture: A review. Cureus 12:e9178. 10.7759/cureus.9178 32802613 10.7759/cureus.9178 PMC7425825

[17] Karatas A, Yilmaz H, Coban G, Koker M, Uz A (2015) The anatomy of circulus arteriosus cerebri (circle of willis): a study in Turkish population. Turk Neurosurg 26(1):54–61. 10.5137/1019-5149.JTN.13281-14.1 10.5137/1019-5149.JTN.13281-14.1 26768869

[18] Kayembe KN, Sasahara M, Hazama F (1984) Cerebral aneurysms and variations in the circle of Willis. Stroke 15:846–850. 10.1161/01.str.15.5.846 6474536 10.1161/01.str.15.5.846

[19] Kedia S, Daisy S, Mukherjee KK, Salunke P, Srinivasa R, Narain MS (2013) Microsurgical anatomy of the anterior cerebral artery in Indian cadavers. Neurol India 61:117–121. 10.4103/0028-3886.111113 23644309 10.4103/0028-3886.111113

[20] Krabbe-Hartkamp M, van de Grond J, de Leeuw FE (1998) Circle of willis: morphologic variation on three-dimensional time-of-flight MR angiograms. Radiology 207(1):103–111. 10.1148/radiology.207.1.9530305 9530305 10.1148/radiology.207.1.9530305

[21] Krishnamurthy A, Nayak SR, Bagoji IB, D’Costa S, Pai MM, Jiji PJ, Kumar CG, Rai R (2010) Morphometry of A1 segment of the anterior cerebral artery and its clinical importance. Clin Ter 161:231–234 20589352

[22] Krzyżewski RM, Tomaszewski KA, Kochana M, Kopeć M, Klimek-Piotrowska W, Walocha JA (2015) Anatomical variations of the anterior communicating artery complex: gender relationship. Surg Radiol Anat 37:81–86. 10.1007/s00276-014-1313-7 24849465 10.1007/s00276-014-1313-7 PMC4295032

[23] Luckrajh JS, Harrichandparsad R, Satyapal KS, Lazarus L (2022) A clinical investigation of the anatomy of the proximal anterior cerebral artery and its association with anterior communicating artery aneurysm. Transl Res Anat 27:100200. 10.1016/j.tria.2022.100200

[24] Murray KD (1964) Dimensions of the circle of Willis and dynamic studies using electrical analogy. J Neurosurg 21:26–34. 10.3171/jns.1964.21.1.0026 14110356 10.3171/jns.1964.21.1.0026

[25] Protection of Personal Information Act (2013) (Act No. 4 of 2013). https://www.gov.za/documents/protection-personal-information-act Accessed 29 May 2025

[26] Sharma S, Krishna H, Dixit SG, Nayyar AK, Khera P, Ghatak S (2023) Systematic review of morphometric analysis of anterior cerebral artery (ACA) emphasizing on its clinical implications. Cureus 15:e37744. 10.7759/cureus.37744 37214049 10.7759/cureus.37744 PMC10193183

[27] Shatri J, Bexheti D, Bexheti S, Kabashi S, Krasniqi S, Ahmetgjekaj I, Zhjeqi V (2017) Influence of gender and age on average dimensions of arteries forming the circle of Willis study by magnetic resonance angiography on kosovo’s population. Open Access Maced J Med Sci 5:714–719. 10.3889/oamjms.2017.160 29104678 10.3889/oamjms.2017.160 PMC5661707

[28] Stats SA (2018) Mortality and causes of death in South Africa: Findings from death notification. https://www.statssa.gov.za/publications/Report-03-08-01/Report-03-08-012018.pdf Accessed 29 May 2025

[29] Tripathi A, Kausar H, Patel AK, Raizaday S, Jain S, Khare S (2021) Retrospective study of variations in anterior communicating artery in human cadaveric brains in Western Uttar Pradesh region. Mædica 16:400. 10.26574/maedica.2021.16.3.400 34925594 10.26574/maedica.2021.16.3.400 PMC8643569

[30] Ujiie H, Liepsch D, Goetz M, Yamaguchi R, Yonetani H, Takakura K (1996) Hemodynamic study of the anterior communicating artery. Stroke J Cereb Circ 27:2086–2094. 10.1161/01.STR.27.11.2086 10.1161/01.str.27.11.2086 8898821

